# Influence of Bio-Based Infill Materials on the Fire Resistance of Panelised Timber Wall Assemblies—A Pilot Study

**DOI:** 10.3390/polym17172420

**Published:** 2025-09-06

**Authors:** Ľudmila Tereňová, Zuzana Vidholdová, Ľubomír Valigurský

**Affiliations:** 1 Department of Fire Protection, Faculty of Wood Sciences and Technology, Technical University in Zvolen, 96001 Zvolen, Slovakia; terenova@tuzvo.sk; 2 Department of Wood Technology, Faculty of Wood Sciences and Technology, Technical University in Zvolen, Masaryka 24, 96001 Zvolen, Slovakia; 3 Faculty of Wood Sciences and Technology, Technical University in Zvolen, Masaryka 24, 96001 Zvolen, Slovakia; xvaligursky@is.tuzvo.sk

**Keywords:** timber wall assembly, bio-based infill material, lignocellulosic materials, sawdust, wood pellets, radiant heat exposure, thermal degradation, fire performance, experimental fire testing

## Abstract

In the pursuit of low-impact and renewable construction materials, various by-products from agriculture, forestry, and the wood processing industry are being explored as potential bio-based infill materials for wall assemblies. This study presents an experimental assessment of the fire performance of timber wall systems composed of block units filled with different lignocellulosic materials, subjected to radiative heat exposure. These assemblies are representative of external walls in contemporary timber-framed buildings. Two configurations were examined: one with sawdust infill and the other with wood pellet infill. Both samples were exposed to radiant heat from the interior side for 60 min, simulating conditions of a fully developed compartment fire. The applied heat flux was 20 kW·m^−2^, delivered by a calibrated radiant panel. The results indicate that even minor design variations—particularly the choice of infill material—can significantly influence the thermal response, degradation kinetics of wood-based components, and the overall fire resistance of the wall assembly. The sawdust-filled system exhibited superior performance, achieving an estimated fire resistance rating of 60 min (60 REI). It showed reduced internal thermal degradation compared to the pellet-filled variant, which experienced greater charring depth due to internal voids between pellets, although it maintained structural integrity.

## 1. Introduction

The increasing demand for low-impact and renewable construction materials has driven growing interest in bio-based and circular approaches within the building sector. Polymers and composites derived from renewable natural sources are intensively investigated for their environmental compatibility, biodegradability, and potential for closed-loop material cycles. Within this framework, wood-based materials represent a significant component of sustainable building technologies [1,2,3,4,5,6,7].

**Figure 1:**
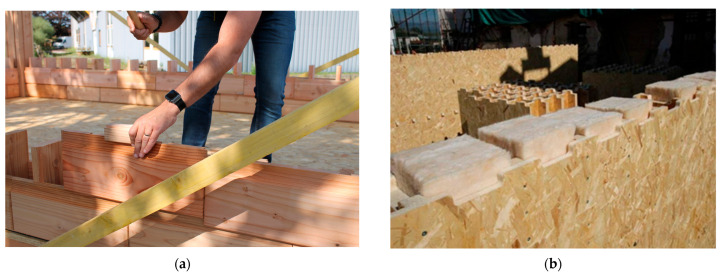
Examples of modern modular timber wall systems using block-based construction: (**a**) Brikawood system composed of interlocking solid wood bricks with an internal cavity that can be filled with natural insulation materials (e.g., sheep wool or mineral wool) [8]; (**b**) XYliving system based on prefabricated modules made from agglomerated wood-based composites, allowing for hybrid wall assemblies with integrated insulation layers [9].

Various modular timber wall systems based on block construction using pre-manufactured wooden units (“wooden bricks”) are being increasingly adopted. These systems represent an eco-friendly, rapid, and precise construction method, often assembled on-site without the use of adhesives or metal fasteners. Structurally, they fall under the category of lightweight timber systems, including post-and-beam, panel, or sandwich-type assemblies. Depending on the design, the configuration may be solid (as in CLT panels) or cavity-based, allowing for the integration of insulation materials. Figure 1a illustrates the Brikawood system, developed by the French company Catharhome. This system enables rapid construction without nails, screws, or glue. It is composed of solid Douglas-fir blocks, whose internal cavities can be filled with wood fibre. Wood fibre provides excellent thermal insulation, with a thermal conductivity coefficient ranging from 0.036 to 0.046 W·m^−1^·K^−1^. The resulting structure offers favourable thermal, mechanical, acoustic, and seismic-resistant properties and is designed to be used without additional cladding or membranes [8]. Figure 1b shows the XYliving system, developed by the French company Saint-Gobain—another prefabricated modular concept, based on agglomerated wood materials [9].

The modular timber wall systems using block-based construction increasingly use natural and recycled infill materials. By-products from wood processing—such as sawdust, wood chips, or recycled wood—are not only in line with the principles of a circular bioeconomy, but also offer advantages in thermal insulation, cost-effectiveness and carbon footprint reduction [10,11,12]. However, their integration into structural assemblies necessitates rigorous evaluation of their behaviour under critical conditions, particularly during fire exposure [13,14,15,16,17]. Although such infill materials deliver multiple environmental and technical benefits, their fire performance must be thoroughly assessed to ensure safe application in building practice [18,19,20,21,22].

In particular, by-products such as straw, hemp, sawdust, or wood shavings are commonly integrated into light timber frame or panelised wall systems to provide thermal insulation, moisture regulation, and carbon sequestration benefits [23,24]. For instance, appropriately treated and protected straw panels have demonstrated enhanced fire resistance [15], with factors such as panel size, shape, and position in the assembly critically influencing fire behaviour. Apte et al. [25] showed that plaster-coated straw bales can withstand intense heat without significant damage, due to the dense structure of the straw combined with a non-combustible casing that limits oxygen ingress and delays ignition. Similar fire performance has been observed for hemp and flax fibres, which, when used as loose fill or in mat form, exhibit delayed ignition and reduced smouldering when combined with protective claddings [23,24,26]. Their porous structure and low volatile content contribute to improved fire stability of wall assemblies.

Another category of bio-based infill materials includes wood-processing residues from sawmills and furniture production—such as sawdust, shavings, and other loose by-products. Sawdust consists of fine, irregular particles that can form a compact fill with minimal internal air voids, influencing both thermal insulation and fire-related properties. Through mechanical densification, these materials can be transformed into wood pellets—a filler form characterized by standardized shape, higher bulk density, and improved mechanical stability. A comparison of key physical and thermal properties of both materials is presented in Table 1.

Compared to loose sawdust, pelletized infill may exhibit different behaviour under fire exposure, particularly in terms of ignition speed, heat transfer, and internal temperature development. Accordingly, evaluating how these two forms of the same base material affect overall fire performance is essential for determining their suitability in wall systems.

Despite the increasing number of research studies on fire safety in wood-based structures, a notable knowledge gap remains regarding the fire resistance of modular timber wall systems filled with unmodified lignocellulosic materials in loose or bulk form. In particular, limited attention has been given to comparing different physical forms of the same base material—e.g., sawdust versus densified wood pellets—with respect to their behaviour under thermal loading and their potential contribution to fire spread.

The aim of this study is to experimentally evaluate the effect of two common wood-based infill materials—sawdust and wood pellets—on the fire performance of panelised timber wall assemblies. The samples were exposed to a controlled radiant heat flux simulating conditions of a fully developed compartment fire from the interior side. To assess the overall fire resistance of the systems, the depth of thermal degradation, the temperature on the unexposed surface, and the structural integrity were monitored. We hypothesize that the geometry and compaction characteristics of the infill materials significantly affect heat transfer and charring behaviour within the wall assemblies. The study addresses the following key research questions:

- How do different types of wood-based infill (sawdust vs. pellets) influence the temperature distribution and thermal insulation performance of OSB-based wall assemblies under radiant heat exposure?
- What are the differences in charring depth and material degradation between the two configurations?
- Can such assemblies achieve the required fire resistance ratings (e.g., REI 60) when filled with renewable, non-synthetic materials?

## 2. Materials and Methods

**Figure 2:**
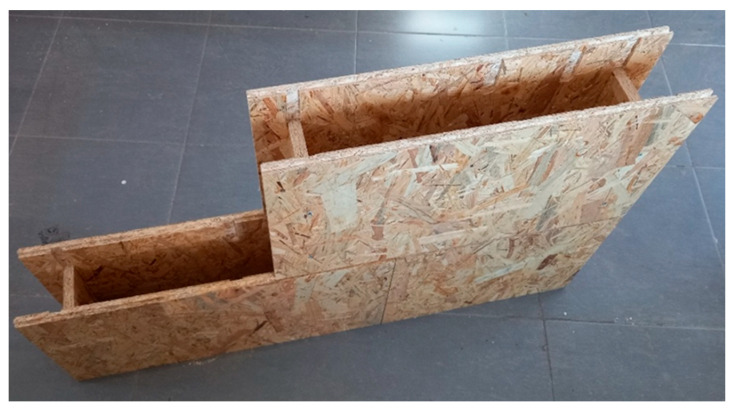
Detail of the core element design showing vertical reinforcement elements inserted into the interior of the OSB blocks to improve the structural integrity and load-bearing capacity of the wall assembly.

To carry out the experiment, we designed a novel construction system based on Oriented Strand Board (OSB), inspired by the timber systems Brikawood and XYliving [8,9], as previously described (see Figure 1). The core element of the system used for testing purposes is a block with dimensions of 137 × 300 × 600 mm and 137 × 300 × 400 mm (width × height × length). Vertical components are inserted inside the blocks to enhance the structural load-bearing capacity of the wall (Figure 2).

### 2.1. Test Materials

Both samples were clad on one side with fire-resistant plasterboard Knauf RED PIANO, 12.5 mm thick, classified as reaction to fire class A2-s1,d0—non-combustible material. The fire-resistant plasterboard was mounted on an OSB frame (KRONOSPAN, Sebes, Romania), 18 mm thick, with a reaction to fire class D-s2,d0—combustible material. The infill materials—sawdust and wood pellets—are classified as reaction to fire class E-s2,d0, also considered combustible (Table 2).

**Figure 3:**
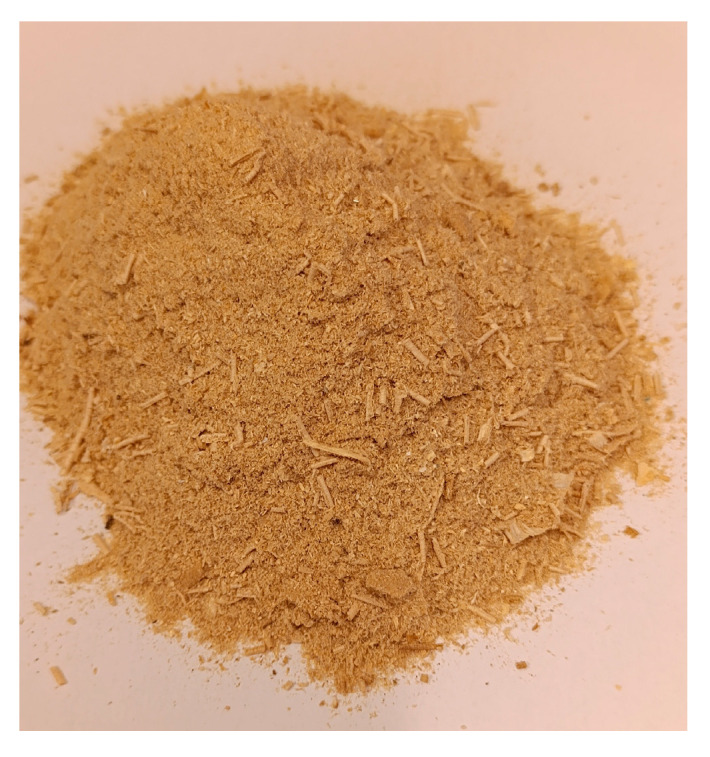
Wood sawdust as the filler.

**Figure 4:**
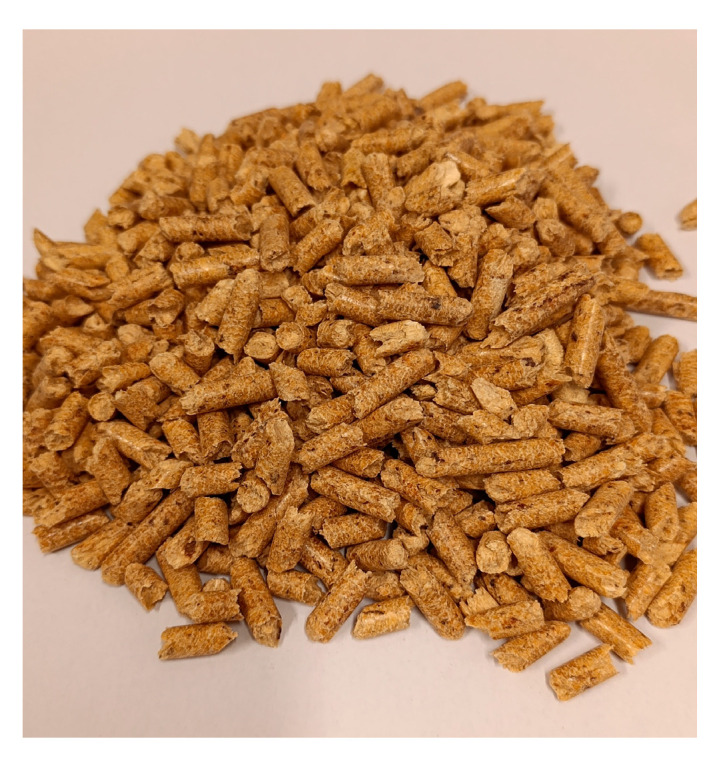
Wood pellets as the filler.

Sample No. 1 was filled with sawdust, predominantly from hardwood species, and also from Particle Boards (PBs) or Medium Density Fibreboards (MDF), and it contains residual glues (Figure 3). Sample No. 2 was filled with wood pellets produced from kiln-dried pine wood, without the use of any chemical additives (Figure 4).

**Figure 5:**
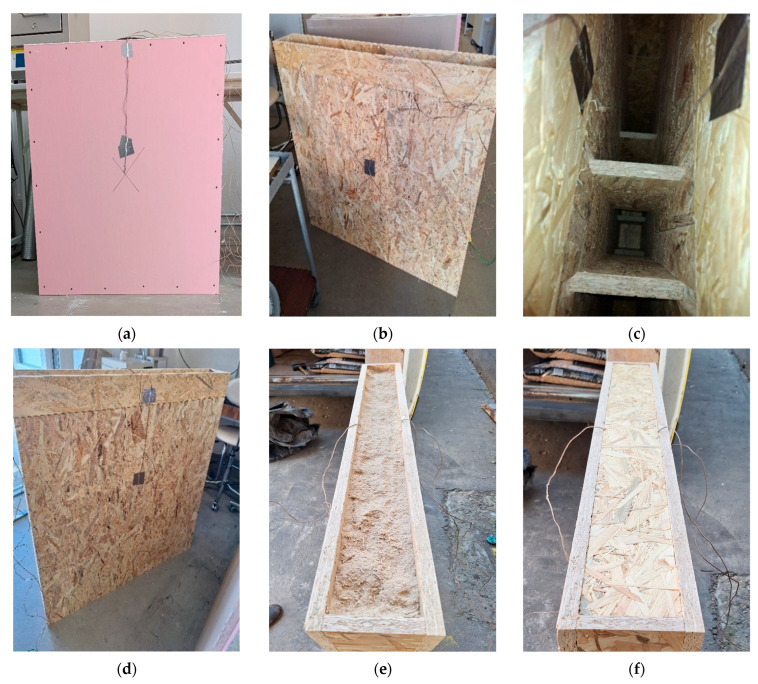
Tested sample: (**a**) Application of the T1 thermocouple on the surface of the plasterboard (internal side of the panel, thermocouple marked with a cross); (**b**) Placement of the T2 thermocouple between the plasterboard and OSB; (**c**) Positioning of the T3 and T4 thermocouples inside the OSB blocks); (**d**) Application of the T5 thermocouple on the surface of the OSB (external side of the panel); (**e**) Filling the cavity with infill material (sawdust or pellets); and (**f**) Sealing the panel with OSB boards.

To monitor the temperature distribution during the test, Ni-Cr thermocouples were installed in the samples prior to sealing. The thermocouples were placed in the central section of each sample, on the interior-facing (T1) and exterior-facing surfaces (T5), as well as at the interfaces between individual layers. Each sample contained five thermocouples, labelled T1 to T5, arranged from the interior to the exterior side (Figure 5).

Although the samples appear visually identical, they differ not only in the type of infill used but also in the method of plasterboard fixation. In Sample No. 1, the plasterboard is attached only along the lateral sides, whereas in Sample No. 2, it is fixed along the entire perimeter (all four sides).

### 2.2. Radiant Heat Exposure Test Method

**Figure 6:**
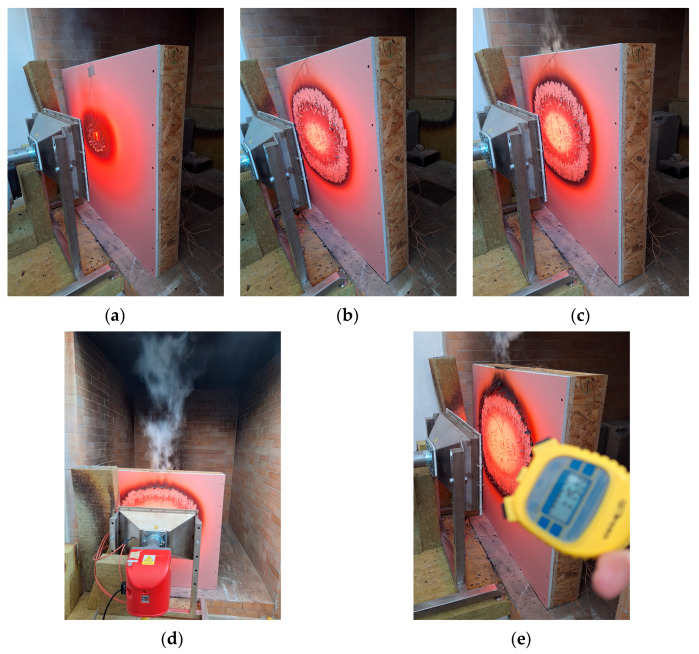
Sample No. 1—sawdust infill: (**a**) at 2:30 min; (**b**) at 16 min; (**c**) at 34 min; (**d**) at 52 min; and (**e**) at 75 min after the start of the test.

The test method is a medium-sized test, which was carried out in a test chamber with dimensions of 1670 mm × 550 mm × 2010 mm (width × depth × height), shown in Figure 6a. The radiant heat source was a radiant panel with dimensions of 500 × 300 mm and a burner output of 20 kW/m^2^. The SGNF 100 burner used is equipped with an automatic SIEMENS LME 41.092C2 (SIEMENS, Munich, Germany) control, powered by 230 V/130 W, with a gas inlet pressure of 30–5 mbar. The fuel is propane butane with automatic regulation of the gas supply to the burner. After 20 min from switching on the device, the temperature of the radiant surface reaches approximately 1000 °C, which corresponds to the maximum thermal conditions of a fully developed compartment fire. The automatic gas control system ensures a constant burner output of 20 kW/m^2^, thus maintaining the required parameters representative of internal fire exposure.

The tested sample structures were placed at a distance of 200 mm from the radiant panel. The chamber was equipped with a system for extracting combustion products (diameter of 250 mm). The measurement took place for 75 min, while the temperatures from the individual thermocouples were automatically recorded using the Almemo 710 measuring device (Ahlborn Mess-und Refge-lungstechnik GhbM, Holzkirchen, Germany) and then stored in the computer.

### 2.3. Thermographic Measurement Procedure

To evaluate the thermal behaviour of the wall panels during radiant heat exposure, infrared thermographic imaging was used to monitor the temperature distribution on the unexposed (exterior) side of each sample. A FLIR thermal imaging camera (model: FLIR C3-X compact thermal camera with thermal sensitivity < 70 mK; FLIR Systems, Inc., Wilsonville, OR, USA) was employed to capture surface temperature gradients during the test. The camera was positioned at a 1 m distance perpendicular to the panel surface, ensuring a full view of the exterior side of the panel. Thermographic snapshots were taken in real-time, with special attention to temperature maxima and the development of heat-affected zones. The measurement results were subsequently analysed and compared for both panel configurations.

## 3. Results

Both tested samples withstood the effects of a simulated internal fire until the 75th minute, at which point testing was concluded. Based on the observed structural behaviour and the recorded temperatures inside and on the external surfaces, the fire resistance of both samples was determined to be 60 min, without failure of the load-bearing capacity (R), integrity (E), or insulation (I) criteria.

### 3.1. Thermal Performance of Sample No. 1—Sawdust Infill

**Figure 7:**
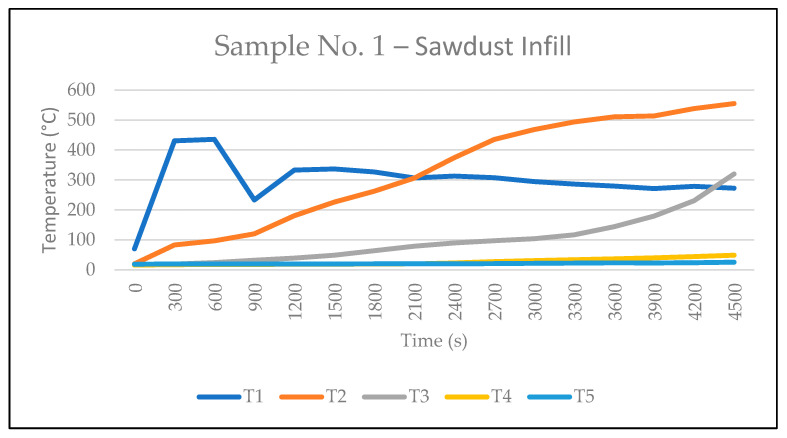
Sample No. 1—Sawdust Infill: temperature development during test.

Figure 6a–c shows the response of Sample No. 1 to radiant heat at 2:30 min, 16 min, and 34 min after the start of the test. At 34 min, smoke began to emerge behind the plasterboard. Temperatures measured by thermocouples are presented in Table 3, and the temperature curve of Sample No. 1 is shown in Figure 7. The 35th minute marked a turning point: at this moment, the temperature recorded by thermocouple T2 exceeded that of T1, indicating accelerated heat penetration into the structure. By the 46th minute, intense smoke was emitted from a gap that had gradually formed between the plasterboard and the OSB panel. This smoke generation was attributed to the thermal degradation of the OSB board when the temperature at T2 reached approximately 435.2 °C. In the 47th minute, a fine crack approximately 100 mm long formed on the plasterboard, indicating a breach of integrity on the interior side of the sample (Figure 6d). By the 52nd minute, very intense smoke emission was observed from the gap between the plasterboard and the OSB, which had widened to about 10 mm, accompanied by a slight bulging of the plasterboard toward the heat source (Figure 6e). The high-level smoke generation was caused by the onset of thermal degradation of the OSB insulation layer, at which the temperature at T2 reached approximately 500 °C, and the temperature at T3 (behind the insulation layer) increased to approximately 117.1 °C. The gap through which smoke escaped intensified due to a missing fixation at the upper edge of the plasterboard.

**Figure 8:**
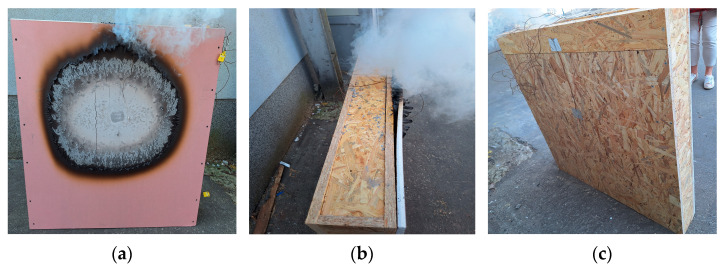

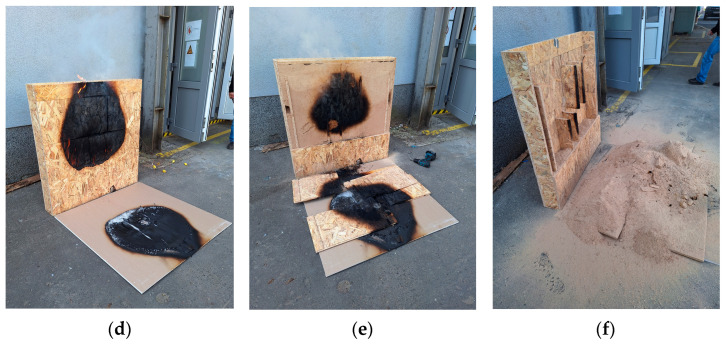
Sample No. 1—sawdust infill after removal from the test chamber and step-by-step disassembly process: (**a**) vertical cracks in plasterboard; (**b**) smoke emission from gap between plasterboard and OSB; (**c**) intact external side; (**d**) degradation of OSB blocks after plasterboard removal; (**e**) partially degraded sawdust infill; and (**f**) partial degradation of vertical OSB internal partitions.

After removal from the test chamber and subsequent disassembly, vertical cracks were observed on the fire-resistant plasterboard (Figure 8a). Smoke was visible in the gap between the plasterboard and the OSB (Figure 8b). Remarkably, the exterior (non-exposed) side remained completely intact (Figure 8c). Removal of the plasterboard revealed degradation of the OSB board (Figure 8d), and beneath the OSB, partial degradation of the sawdust infill was observed (Figure 8e). Additionally, partial charring of the internal vertical OSB partitions was also evident (Figure 8f).

### 3.2. Thermal Performance of Sample No. 2—Pellets Infill

**Figure 9:**
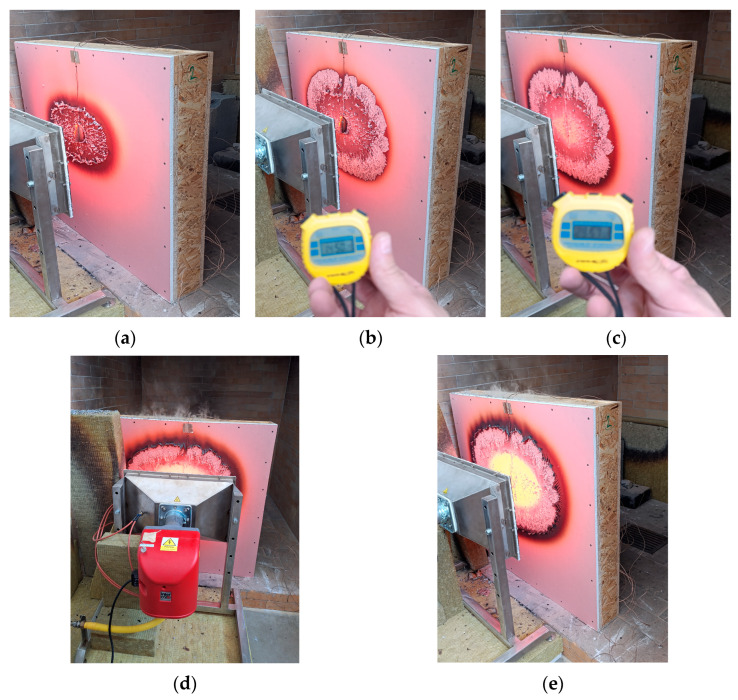
Sample No. 2—pellet infill: (**a**) at 2:30 min; (**b**) at 15 min; (**c**) at 30 min; (**d**) at 52 min; and (**e**) at 73 min after the start of the test.

Figure 9 shows Sample No. 2 at 2:30 min (a) and 15 min (b) after the start of the test. At 30 min, no deformation or smoke emission was observed, likely due to the different method of plasterboard fixation along the entire perimeter (i.e., on all four sides), which preserved the integrity of the panel throughout the test.

**Figure 10:**
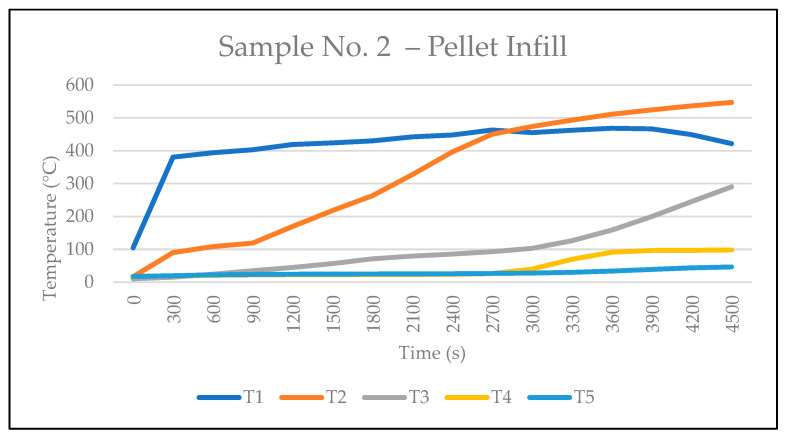
Sample No. 2—pellet infill: temperature development during test.

The temperature data recorded by the thermocouples for Sample No. 2 are presented in Table 4, while Figure 10 shows the corresponding temperature curve. At the 47th minute, the temperature measured by thermocouple T2 surpassed that of T1. This significant change occurred 12 min later than in Sample 1, primarily because the plasterboard remained intact throughout the test, being securely fixed around the entire perimeter of the sample. Concurrently, light smoke was observed escaping between the OSB elements at the rear of the sample.

From the 49th min, smoke began to escape through the upper layers, and between minutes 64 and 70, smoke emission intensified significantly. At 73 min, smoke was also observed escaping from the rear gap between the pellet infill and the OSB, indicating that the pellet layer had degraded through its full thickness and that the rear wall of the OSB panels had begun to degrade as well. The temperature recorded at thermocouple T4 reached 98.2 °C, almost 50 °C higher than in Sample No. 1. Although the intact plasterboard delayed the moment when the temperature at T2 exceeded that at T1, subsequent heat transfer into the interior of the structure was more intense due to the higher density of the wood pellets compared to sawdust [27,28,29]. Consequently, the thermal conductivity coefficient of the pellets is higher (λ = 0.15–0.19 W·m^−1^·K^−1^, Table 1, refs. [30,31,32]), which further promoted heat penetration into the assembly. Unlike sawdust, which provided better insulation owing to its lower density and thermal conductivity, the pellet infill facilitated more rapid heating of the underlying layers. No cracks formed on the interior plasterboard surface throughout the test duration.

**Figure 11:**
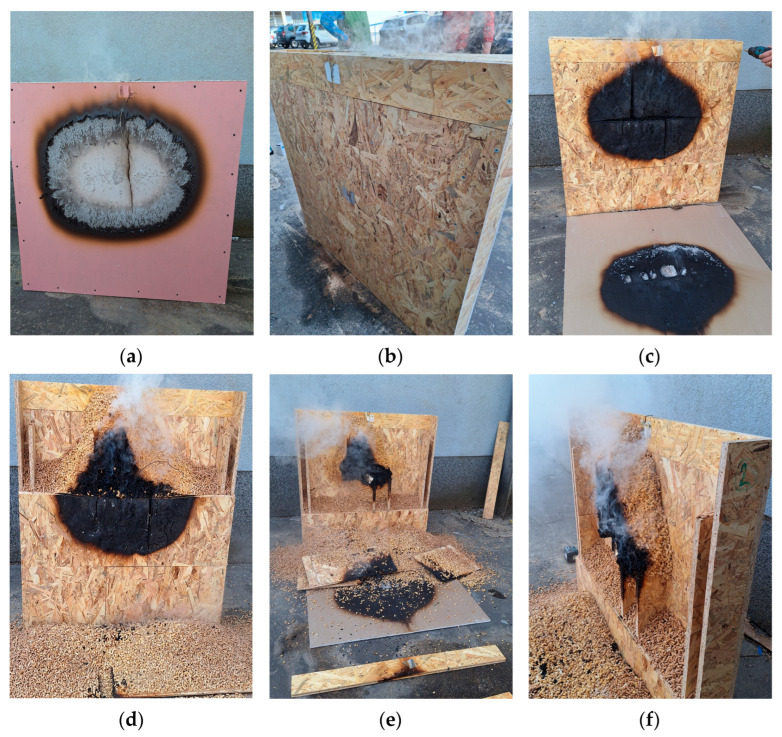
Sample No. 2—pellet infill: after the test and step-by-step disassembly process: (**a**) exposed side of the plasterboard, (**b**) non-exposed side (**c**) degradation of OSB blocks under plasterboard; (**d**) degradation of the pellet insulation layer; (**e**) release of the internal pellet infill; and (**f**) fused pellets and degraded vertical OSB internal partitions.

Figure 11 shows Sample No. 2 after testing. A visible crack appeared on the plasterboard on the exposed interior side (Figure 11a), while the non-exposed exterior side remained completely intact (Figure 11b). Further image shows significant degradation of the OSB blocks (Figure 11c), as well as thermal damage to the pellet insulation layer (Figure 11d). Degraded and fused pellets adhering to the OSB board were visible on the rear side of the sample (Figure 11e,f), along with discoloured OSB material in the heat-exposed area.

### 3.3. Thermographic Analysis of the Unexposed Side

**Figure 12:**
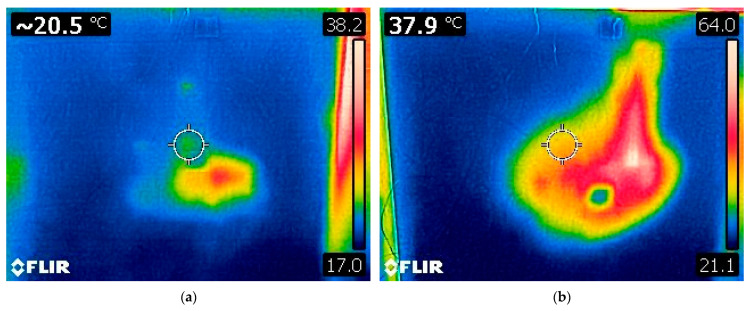
Thermographic images showing surface temperature distribution on the exterior (unexposed) sides of the wall panels during radiant heat exposure: (**a**) Panel with sawdust infill (sample No. 1), maximum temperature 38.2 °C. (**b**) Panel with pellet infill (sample No. 2), maximum temperature 64.0 °C. The circle indicates the location where the surface temperature was measured.

The thermographic images (Figure 12) represent the temperature distribution on the exterior (unexposed) sides of the tested wall panels during radiant heat exposure.

In the case of the panel with sawdust infill (Sample No. 1), the thermal image shows localized heat accumulation, with a maximum surface temperature of 38.2 °C. Most of the surface remains within the blue-to-green range (17–25 °C), indicating effective thermal insulation and limited heat transfer through the panel. The small, concentrated heat zone suggests delayed and restricted thermal progression, which corresponds to the compact nature of the sawdust infill.

In contrast, the panel with pellet infill (Sample No. 2) exhibits a larger and more intense heat-affected zone, with surface temperatures reaching up to 64.0 °C. The wider red and white areas indicate greater heat penetration and less effective insulation performance. This aligns with the overall experimental results presented in Section 3.2, where the pellet infill enabled faster and deeper thermal degradation due to air gaps and increased oxygen circulation within the material.

These thermal images visually support the conclusion that sawdust provides superior insulation and thermal resistance, while pellet infill is more prone to transmitting heat, thus confirming the influence of infill morphology on the fire performance of modular timber panels.

## 4. Discussion

The experimental results revealed that the panel with sawdust infill (Sample No. 1) exhibited more favourable performance and greater resistance to heat under simulated internal fire conditions. The improved fire resistance was primarily attributed to the type of infill material used. The sawdust displayed a compact character that limited oxygen availability and slowed the decomposition of the material, resulting in a degradation depth of only 20 mm. In contrast, the panel with pellet infill (Sample No. 2) allowed for more substantial heat transfer due to the higher density of the pellets and increased oxygen reactivity within the air gaps of the pellet layer under thermal load. Upon dismantling, the pellet layers were found to be charred to a depth of approximately 40 mm. Ultimately, thermal degradation extended through the full thickness of the insulation, with charred and fused pellets adhering to the rear OSB panel in the heat-exposed zone, accompanied by visible panel discolouration. These results clearly demonstrate that the morphology of the infill material plays a key role in thermal performance, as observed in previous studies on other bio-based materials such as straw and hemp [15,23,24,25,26]. Due to its compact and cohesive structure, lower density, and lower thermal conductivity (Table 1, refs. [27,28,29,30,31,32]), sawdust reduced internal heat transfer and limited charring depth. In contrast, the interstitial air gaps in the pellet infill promoted convective heat transfer and oxygen flow, thereby reducing thermal insulation efficiency under fire exposure.

The infill material had a direct impact on heat transfer during thermal loading in both panels. In the sawdust-filled panel, smoke emission began at 34 min into the test, coinciding with the temperature recorded at thermocouple T2 (315 °C) exceeding that at T1 (303 °C) (Table 3). In the pellet-filled panel, this transition occurred at 47 min, when the temperature at T2 reached 465.5 °C, surpassing T1 at 459.3 °C (Table 4). These results were supported by thermographic imaging (Figure 12), which visualized the thermal differences. The thermal response variations between the panels underscore the influence of material morphology on temperature distribution. Although smoke emission in the sawdust-filled panel occurred earlier, the lower maximum temperatures recorded suggest superior thermal insulation and greater stability.

In addition to the infill material, the composition of the tested samples was influenced by the use of OSB panels. While OSB is classified as a combustible material with a fire reaction class of D-s2, d0 (Table 2, ref. [34]), it plays a crucial structural role in panelised timber assemblies. Prior research [22,35,36] has demonstrated that fire performance can be significantly improved by selecting appropriate materials and arranging layers effectively. In particular, placing an OSB base board beneath a fire-protective cladding such as plasterboard can delay the onset of thermal degradation. Moreover, the presence of a suitable insulation layer behind the OSB contributes to further reducing heat transfer into the assembly, thereby preserving structural integrity.

The construction design—particularly the fixation details—also influenced the observed results. In Sample No. 1, the plasterboard was fastened only along the vertical edges, creating a gap at the top during heating. This discontinuity allowed smoke leakage around the 34th minute. In contrast, in Sample No. 2, the plasterboard was secured along the entire perimeter, delaying smoke emission until around the 50th minute. This methodological inconsistency must be acknowledged as a limitation of the pilot study, as it introduced a structural variable that may have contributed to the performance differences between the two assemblies. Nevertheless, the findings remain valuable, highlighting the interaction between infill material and construction detailing. A shared advantageous design feature was the inclusion of vertical timber blocks within the modular panels. The staggered arrangement of these partitions contributed to reducing internal heat transfer and slowing fire propagation. Upon dismantling, localized degradation was observed at the partition edges—up to 20 mm in Sample No. 1 and up to 40 mm in Sample No. 2. These findings illustrate how bio-based infill interacts with internal structural components, offering insights into design strategies for improved fire resistance. Future investigations should apply standardized fixation methods across test specimens to isolate the effect of the infill material from other constructional variables.

A noteworthy observation was that throughout both tests, the plasterboard integrity remained uncompromised. Only Sample No. 1 exhibited a minor surface crack at 47 min. However, both samples met the E (integrity) fire resistance criterion on the unexposed side for the entire test duration. The I (insulation) criterion was also satisfied, as limit temperatures on the unexposed surfaces were not exceeded. At the conclusion of the test, surface temperatures on the unexposed side were 25.8 °C for the sawdust-filled panel and 46.7 °C for the pellet-filled panel. These results confirm that both types of modular timber assemblies, when properly designed, can meet essential fire performance requirements. Despite the differences in thermal behaviour, both infill types helped maintain insulation and integrity criteria under radiant heat exposure.

Comparable findings were reported by Tereňová et al. [37], who investigated natural thermal insulation materials in timber-frame wall assemblies. The tested panels comprised KVH timber framing with OSB on both sides and internal plasterboard cladding. One panel used wood fibre insulation, and the other hemp fibre. The same medium-scale radiant heat test method was applied. The hemp-filled panel exhibited faster temperature increase and lower thermal resistance compared to the panel filled with pressed wood fibre. Both panels achieved an estimated fire resistance of 60 min (REI 60). This aligns with the present study’s conclusion that the morphology and thermal properties of renewable, non-synthetic materials—such as sawdust or wood fibre—can support achieving high fire resistance ratings.

Additional relevant results were presented by Boráros and Gašpercová [38], who examined the thermal degradation of various insulation materials—including expanded polystyrene (EPS), wood fibre, hemp, and recycled textile—under radiant heat exposure. The insulation specimens (500 × 500 × 100 mm), mounted in aluminium frames and covered with plasterboard, were placed 50 mm from the radiant panel for a 30 min test. Due to the short distance, plasterboard integrity was compromised within the first few minutes. Recycled textile insulation exhibited the best performance, with melting only at higher temperatures (506 °C) and no sustained combustion after the heat source was removed. The maximum degradation depth was limited to 40 mm. Wood fibre and hemp also performed relatively well; however, wood fibre continued to burn after heat removal, while hemp self-extinguished. Maximum temperatures reached 450 °C and 475 °C, with degradation depths of 70 mm and 100 mm, respectively.

These findings suggest that wood fibre, hemp, and recycled textile insulation may be viable alternatives for fire-resistant timber panel assemblies similar to those tested in this study.

Taken together, the results of this and related studies demonstrate the potential of renewable, non-synthetic materials to meet REI 60 fire resistance requirements—especially when combined with appropriate panel construction methods and detailing.

## 5. Conclusions

The conducted experiments confirmed that both tested panel samples—the one with sawdust infill and the one with pellet infill—met the requirements for an estimated fire resistance rating of 60 min, fulfilling the E (integrity) and I (insulation) fire resistance criteria. The R criterion (load-bearing capacity and structural stability) can only be determined through full-scale fire tests, in which the construction is installed under real building conditions.

The type and morphology of the infill material directly influenced temperature distribution, thermal insulation performance, and the extent of material degradation under radiant heat exposure. The panel filled with sawdust exhibited superior thermal resistance, with a maximum charring depth of 20 mm. This was attributed to its compact structure, which limited oxygen flow and slowed thermal decomposition. In contrast, the pellet-filled panel showed deeper thermal degradation (up to 40 mm) and greater heat penetration due to air gaps between the pellets, which facilitated convective heat transfer and increased material reactivity.

These observations confirm that the morphology of renewable infill materials significantly affects fire performance, thereby answering the first two research questions related to heat transfer and charring behaviour.

Despite the observed differences in thermal behaviour, both panels fulfilled the E and I criteria throughout the test duration. This demonstrates that modular timber wall assemblies filled with renewable, non-synthetic materials can achieve satisfactory fire resistance ratings—such as 60 REI—when properly designed and detailed. These findings directly address the third research question and underscore the viability of sustainable, bio-based insulation in fire-safe timber construction.

While wood pellets offer practical advantages such as easier handling and higher bulk density, sawdust proved more effective in limiting thermal degradation and is thus better suited for applications requiring enhanced fire resistance.

Overall, the composition of the tested panels—including the layer arrangement and the presence of internal vertical partitions—played a critical role in resisting radiant heat exposure. According to current Slovak building regulations, the tested construction systems are suitable for use in timber buildings up to three above-ground storeys, supporting their potential application in sustainable low- and mid-rise wood architecture.

## Figures and Tables

**Table 1:** Comparative Physical, Thermal, and Application Properties of Sawdust and Wood Pellets.

Property	Sawdust	Wood Pellets	Source(s)
Particle Morphology	Irregular, fine-to-medium-size flakes	Cylindrical (Ø 6–8 mm; length 10–30 mm)	[27]
Bulk Density [kg·m^−3^]	Low (~120–300)	High (~600–1000)	[27,28,29]
Thermal Conductivity λ [W·m^−1^·K^−1^]	0.04–0.07 (typically ~0.048)	0.15–0.19	[30,31,32]
Porosity [%]	High (~80%)	Moderate to low (~30–60%)	[29]
Moisture Content [%]	8–15 (variable; depends on source and storage)	6–10 (standardized)	[27]
Heat of Combustion [MJ·kg^−1^]	5–17	15–19	[28]
Specific Heat Capacity [J·kg^−1^·K^−1^]	~1822	~1074–1253	[32,33]
Ignition Characteristics	Quick ignition due to high surface area	Slower ignition, but more stable combustion	
Processing Requirements	Minimal (by-product, unprocessed)	Requires drying, grinding, pelletizing	[27]
Handling Characteristics	Dusty; prone to moisture absorption	Clean and compact; easier to handle	[27]
Common Applications	Insulation, compost, bio-composite panels, lightweight fillers	Fuel, bio-composite filler, compressed panels	[27]
Thermal Performance (as Insulator)	Better insulator due to lower λ and higher porosity	Less effective insulator, but retains heat well	[28,30,31,32]
Environmental Impact	Low (utilization of waste material)	Higher, due to energy-intensive processing	[27]

**Table 2:** Comparative Properties, Applications, and Fire Classification of Selected Wood-Based Materials.

Material	Advantages	Applications	Density (kg·m^−3^)	Reaction to Fire Class *
OSB 3 (Kronospan)	- Moisture resistant - High stiffness and - load-bearing capacity - Good dimensional stability - Recyclable	- Wall, floor, and roof sheathing - Furniture frames - Interior partitioning	≥600	D-s2, d0 (combustible)
RED PIANO (GKF) Knauf	- Excellent acoustic insulation - Flexible and easy to install - Optimized dimensions for handling	- Partition walls and suspended ceilings - Fire-resistant cladding of structural elements	≥680	A2-s1, d0 (non-combustible)
Wood sawdust	- Easy application - Good thermal insulation properties - Recyclable	- Raw material for particleboard - Infill insulation material	<300	E-s2, d0 (combustible)
Wood pellets	- 100% natural and renewable - Free of chemical additives	- Fuel for boilers and fireplaces - Alternative use as infill material	≥600	E-s2, d0 (combustible)

Note: * Fire classification: A2—Non-combustible materials; D, E—Combustible materials; Degree of smoke production (s1 = low, s2 = high); Presence of flaming droplets (d0 = none).

**Table 3:** Sample No. 1—Sawdust Infill: Measured Temperatures (°C).

Time (s)	T1	T2	T3	T4	T5
0	70.40	20.50	18.20	16.10	19.20
300	430.40	83.30	19.40	17.40	19.90
600	435.80	97.10	24.60	18.00	19.80
900	233.50	120.20	32.50	18.30	19.70
1200	333.20	180.70	39.50	18.60	19.80
1500	336.70	226.20	49.10	19.00	19.80
1800	326.60	262.40	64.20	19.40	20.10
2100	306.60	306.40	78.90	20.20	20.20
2400	312.90	374.50	89.70	23.20	20.40
2700	307.40	435.20	97.50	27.60	21.00
3000	294.70	468.20	104.10	31.00	21.80
3300	286.20	493.70	117.10	34.00	22.70
3600	279.50	510.70	144.20	36.80	23.50
3900	271.20	513.40	179.50	40.20	23.20
4200	278.60	538.00	230.90	44.40	24.20
4500	272.60	554.90	320.20	49.30	25.80

**Table 4:** Sample No. 2—pellet infill: measured temperatures (°C).

Time (s)	T1	T2	T3	T4	T5
0	104.70	17.00	10.20	18.10	17.50
300	380.80	90.20	15.30	19.40	19.90
600	393.90	108.40	24.20	20.90	22.20
900	403.20	119.00	35.10	22.10	23.40
1200	419.10	169.70	44.70	22.60	24.10
1500	424.00	218.10	56.50	22.60	24.60
1800	430.20	263.30	70.90	22.90	25.00
2100	442.20	327.50	79.60	23.20	25.60
2400	448.00	396.50	85.60	23.80	25.90
2700	463.10	450.20	92.90	26.20	26.60
3000	455.40	474.10	103.20	39.70	27.80
3300	462.60	493.30	126.10	69.50	30.00
3600	468.90	511.30	158.50	91.30	33.70
3900	466.80	524.60	199.70	96.60	38.90
4200	449.30	536.50	245.30	97.00	43.80
4500	421.70	547.40	290.40	98.20	46.70

## Data Availability

The original contributions presented in this study are included in the article. Further inquiries can be directed to the corresponding authors.
